# Candidate treatments for long COVID: a narrative review of expert and patient-driven priorities

**DOI:** 10.3389/fmed.2026.1734600

**Published:** 2026-02-27

**Authors:** Shaira Nicole Baptista, Tiffany Atkins, Samantha Chakraborty, Mina Bakhit, Paul Glasziou, Oyungerel Byambasuren

**Affiliations:** 1 Australian Living Evidence Collaboration, Monash University School of Public Health and Preventive Medicine, Melbourne, VIC, Australia; 2 Bond University Institute for Evidence-Based Healthcare, Robina, QLD, Australia

**Keywords:** living evidence, long Covid, narrative review, post-acute sequalae of SARS-CoV-2 infection, research prioritisation

## Abstract

**Objective:**

To map the existing evidence for candidate treatments for long COVID that were prioritised by clinicians and people with lived experience, and to characterise their feasibility, acceptability and safety.

**Study design:**

The study was conducted as a narrative review using pragmatic methods including iterative stakeholder-informed decision-making a monthly-updated evidence search, rapid lay evidence summaries and a structured research prioritisation process.

**Data sources:**

Potential candidate treatments were identified via a combination of database and trial registry searches. These were then ranked by clinicians and people with lived experience using surveys. Evidence summaries for the top 14 interventions (low-dose naltrexone, antivirals, metformin, nicotine, vagus nerve stimulation, antihistamines, guanfacine, colchicine, nattokinase, intravenous immunoglobulins, monoclonal antibodies, coenzyme Q10, multicomponent rehabilitation packages, and exercise training) were created. Prioritised treatments were collated first by searching a collaborative living evidence database (updated monthly) of relevant systematic reviews and randomised controlled trials and then by conducting supplementary searches of other study designs.

**Data synthesis:**

Six of 14 interventions had long-COVID-specific randomised controlled trial (RCT) evidence (exercise [16 RCTs], multicomponent packages [5 RCTs], coenzyme Q10 [2 RCTs], antivirals [1 RCT], vagus nerve stimulation [1 pilot RCT], monoclonal antibodies [1 small RCT]); the remainder relied on indirect or very low-certainty data (e.g., uncontrolled studies or mechanistic rationale). Across interventions, evidence certainty was mostly low to very low, and safety/feasibility varied.

**Conclusion:**

This review prioritises and maps candidate treatments for long COVID. There was insufficient direct evidence to inform clinical recommendations. Rather, the treatments presented in this review represent those that could be rigorously tested in clinical trials as they show biological plausibility and/or are feasible and acceptable to people with lived experience and clinicians.

**Registration:**

A review protocol was not prospectively registered because the review adopted an iterative approach to support priority setting rather than clinical guidance.

## Introduction

Long COVID, also known as post-acute sequelae of COVID-19 (PASC), is a significant health concern affecting millions of people globally (1). Long COVID is defined as symptoms persisting for more than 3 months from the onset of SARS-CoV-2 infection, lasting at least 2 months and not explained by an alternative diagnosis (2) and poses considerable challenges for healthcare systems in Australia and around the world with associated economic and social burdens (3). In addition to being a relatively recent condition, long COVID can present in heterogeneous ways, and there are no clear treatment targets or diagnostic tests (4, 5). Consequently, despite the growing prevalence of this condition, there is a lack of evidence for effective treatments and recent efforts to map care available for long COVID in Australia have highlighted significant gaps in service delivery (6–9). As a result, clinicians and people living with long COVID may resort to unproven or off-label therapies in search of relief (10). This situation demands immediate attention because the use of interventions without robust evidence raises serious ethical and safety concerns.

Currently, there are a number of ongoing initiatives that are investigating the efficacy of a wide range of potential long COVID treatments ranging from existing medications for other conditions to novel therapeutic approaches (11). Although previous reviews investigate the effectiveness of various long COVID treatments, they do not adequately address which treatments are most promising based on their feasibility, safety and acceptability (9). Furthermore, given the extensive and evolving number of potential treatments for long COVID, there remains a critical gap in synthesising the evidence in a way that not only reflects the clinical efficacy and biological plausibility, but also incorporates the priorities of both clinicians and people with lived experience of long COVID. Including the perspectives of people with lived experience and clinicians is important to ensure that long COVID treatments are not only effective, but also acceptable to people with lived experience and applicable in a clinical setting. Furthermore, people living with long COVID experience a wide range of symptoms that vary in severity and impact (12). Integrating their perspectives is important to reflect the real-world complexities of living with the condition. Finally, treatments that align with the values and preferences of people with lived experience are more likely to be adhered to, potentially improving their effectiveness (11). The aim of this review was therefore to summarise the current evidence for effectiveness, safety, feasibility, and acceptability for treatments prioritised by clinicians and people with lived experience of long COVID to help inform future research.

## Methods

Potential interventions to treat long COVID were identified through a multi-component process which is described elsewhere. In brief, it involved systematic literature searches (until January 2025–see Appendix A in Supplementary material) (13) and ranking of long COVID treatments by people living with long COVID and clinicians who have a special interest in long COVID. Evidence summaries were then written for the final top 14 ranked interventions.

### Search and study selection

The evidence presented in each summary was collated based on a hierarchical approach. We aimed to first identify relevant SRs and then RCTs from our previous literature searches. If no systematic reviews or RCTs were identified in our library, we conducted a supplementary search to identify evidence from other study designs (e.g., cohort studies, case–control studies). This search was conducted across PubMed, Cochrane Library, Embase and Epistemonikos. We used simple, broad search terms such as the “intervention of interest” AND “long COVID.” This approach was chosen given the heterogeneity of long COVID, the diversity of potential interventions and the very early-stage evidence base.

Studies were selected based on their relevance to the research question and their methodological quality. The selection process was conducted independently by two reviewers, with disagreements resolved through discussion or consultation with a third reviewer. If a systematic review was identified, this was presented as the primary evidence due to their high methodological rigor and ability to establish causal relationships. Any RCTs not included in the systematic review were added in narratively. In the absence of systematic reviews and RCTs, we included non-randomised and observational studies.

The eligibility criteria were as follows:

Population: Adults and adolescents meeting the operational definition of long COVID.
Interventions: Shortlisted candidate treatments.
Comparators: Any (including usual care, sham, no treatment).
Outcomes: Patient-reported and objective measures including fatigue, function, quality of life, cognitive outcomes, post-exertional symptom exacerbation, and safety.
Study designs: Prefer SRs and RCTs; if absent, include non-randomised and observational studies.
Acute COVID treatment studies without long-COVID follow-up, editorials, animal models and in-vitro studies were excluded, but used to explain potential mechanisms of action.

### Generation of evidence summaries

Key information including a brief rationale for the treatment, a summary of the key evidence as well as available safety, feasibility, and acceptability information for the Australian context was summarised into a pre-determined template. The quality of the evidence base was rated to help describe the level of evidence available for each treatment. For example, treatments that had several RCTs underpinning them were rated as high, whereas those that had only case studies were rated as very low. We did not conduct formal risk-of-bias or GRADE assessments, as the purpose of this study was to develop an accessible evidence summary for a general audience. Feasibility included availability in Australia, regulatory status, PBS listing, cost and resource considerations. Acceptability reflected patient and/or clinician willingness, adherence considerations and side-effect and other patient burden (14). In addition, any relevant ongoing trials identified from clinical trial registries^1^,^2^ were noted and briefly described to provide context for future research in the field.

## Results

Fourteen intervention summaries were created based on the prioritisation process. Table 1 summarises each of these while Appendix 1 in Supplementary material contains each included evidence summary in detail. The available information about treatment effectiveness, acceptability and feasibility are presented in the order in which they were ranked by people with lived experience and clinicians.

**Table 1:** Summary of 14 long COVID Interventions identified as being most relevant.

Intervention name	Potential mechanism of action	References	Evidence quality	Summary of Evidence for effectiveness	Safety and feasibility in the Australian context
Low Dose Naltrexone (LDN)	Reduction of viral replication and persistence	Bonilla et al. (16), Isman et al. (17), O'Kelly et al. (18), Tamariz et al. (19)	Low	Possible small benefit	Safe and inexpensive
			4 non-randomised pre post studies		
Antivirals	Reduction of viral replication and persistence	Geng et al. (21)	Moderate	No statistically significant improvement in symptoms.	Safe with mild side effects. Can interact with other medicines. Restricted access in Australia
			1 RCT		
Antihistamines	Mast cell and histamine pathway inhibition	Salvucci et al. (34)	Very low	May be effective, but very low quality evidence	Usually safe with minimal side effects. Fairly inexpensive and accessible
			1 non-randomised study		
Nicotine	Receptor-mediated neuromodulation and inflammation reduction	Kloc et al.(28), Leitzke et al.(29)	Very low	Unclear	Rare serious side effects and relatively inexpensive
			1 case series		
Metformin	Mitochondrial, metabolic, inflammatory, and vascular modulation.	(23–26)	None	Unknown	Safe with minimal side effects and relatively Inexpensive
			0 studies		
Vagus Nerve Stimulation	Autonomic and inflammatory regulation	Khan et al. (31), Badran et al. (32)	Low	Possible small benefit	Safe and relatively inexpensive
			1 pilot RCT and 1 non-randomised study		
Guanfacine	Neuroinflammation reduction and cognitive network support	Arnsten et al. (36), Fesharaki Zadeh et al. (37), Fesharaki-Zadeh et al. (38)	Very low	Unclear	Fairly safe and relatively inexpensive
			1 case series		
Colchicine	Innate immune and inflammasome suppression; endothelial and anti-fibrotic effects	Reyes et al.(39), Leung et al. (40), Deftereos et al. (41)	None	Unknown	Safe with careful consideration of dosage and relatively inexpensive
			0 studies		
Monoclonal antibodies	Viral clearance and immune modulation	Proal et al. (51), Gaylis et al. (52)	Low	Probably no benefit	Safe in the short term but limited data on long-term safety. Not accessible for Long COVID in Australia
			1 pilot RCT and 1 case series		
Nattokinase	Microclot and spike protein degradation (*in vitro*)	Grixti et al. (42), Tanikawa et al. (43)	None	Unknown	Considered safe and relatively inexpensive
			0 studies		
Intravenous immunoglobulin (IVIg)	Immune response	McCarthy et al. (45), Hogeweg et al. (46), Thompson et al. (48)	Very low	Unclear	Considered safe but not available as a Long COVID treatment in Australia
			1 case series and 2 retrospective case–control studies		
Coenzyme Q10	Mitochondrial energy and antioxidant support	Bonakdar and Guarneri (55), Hansen et al. (56)	Moderate	Probably no effect	Considered safe and relatively inexpensive
			2 RCTs and 1 observational study		
Multicomponent intervention package (may include symptom management, pacing, sleep regulation and psychological support)	Multisystem functional rehabilitation	Kuut et al. (59), Leon-Herrera et al. (60), McGregor et al. (62), Espinoza-Bravo et al. (68), Jimeno-Almazan et al. (69), Kaczmarczyk et al. (71), Kerling et al. (72), Lai et al. (75), Mooren et al. (76), Pleguezuelos et al. (77), Pleguezuelos et al. (78), Ramirez-Velez et al. (79), Sick et al. (80)	High	Possible small benefit	Generally safe especially when provided under supervision
			5 RCTs		Moderately expensive
Exercise training	Cardiopulmonary and functional adaptation	McGregor et al. (62), Sanchez Mila et al. (64), Bai et al. (65), Barzet al. (66), Cunha et al. (67), Espinoza-Bravo et al. (68), Jimeno-Almazan et al. (69), Jorge et al. (70), Kaczmarczyk et al. (71), Kerling et al. (72), Lai et al. (75), Mooren et al. (76), Pleguezuelos et al. (77), Pleguezuelos et al. (78), Ramirez-Velez et al. (79), Sick et al. (80), Gloeckl et al. (81)	High	Possible benefit	Quite safe especially if supervised
			16 RCTs		Can be inexpensive

RCT, Randomised controlled trial; PBS, Pharmaceutical Benefits Scheme.

### Low dose naltrexone

Low Dose Naltrexone (LDN) may improve long COVID symptoms by acting on underlying inflammatory and pain pathways (15). There are no published RCTs or systematic reviews that specifically report on the effectiveness of LDN on long COVID, however there are three ongoing clinical trials investigating the effectiveness of LDN for fatigue and other improvements in a variety of long COVID symptoms. Four non-randomised pre–post studies evaluated low-dose naltrexone (LDN), alone or in combination with Nicotinamide Adenine Dinucleotide (NAD+), across dosing ranges from 0.5 to 6 mg/day. Pooled results favoured LDN over no treatment across all reported outcomes, including fatigue, pain, brain fog, sleep quality, and daily functioning. However, the authors rated the evidence as low quality because of considerable risk of bias (16–19). LDN is relatively safe with minimal side-effects, although a small number of people experience some side effects including headaches and sleep difficulties (20). LDN is relatively accessible in Australia and can be prescribed by a general practitioner.

### Antivirals

Antivirals (Nirmatrelvir, Molnupiravir, and Remdesivir) may help treat the underlying pathophysiology of long COVID by limiting viral replication, potentially targeting persistent SARS-CoV-2 reservoirs that may cause ongoing long-term symptoms. The best available evidence comes from a moderate-quality RCT of 155 participants which found no significant improvement between patients treated with Paxlovid (300 mg of nirmatrelvir with 100 mg of ritonavir) twice a day for 15 days and those who received a placebo, after 15 weeks (21). Paxlovid is generally considered safe, with most side effects being mild (such as altered taste and diarrhea), but it can interact with other drugs and must be used only under medical supervision because of potentially serious side effects such as allergic reactions, liver problems as well as headaches, nausea, stomach pain and high blood pressure (22). Feasibility is limited in Australia, where access is restricted by strict prescribing criteria, high cost without subsidy, and the unavailability of the version of Paxlovid used in clinical studies. Currently, eight clinical trials are ongoing internationally, testing various antivirals (including Paxlovid™, amantadine, ensitrelvir, and others) and various durations which may provide more reliable evidence about the efficacy of Antivirals as a treatment to improve a variety of symptoms of long COVID such as fatigue, brain fog, shortness of breath, and body aches.

### Metformin

Metformin is being considered as a potential treatment for long COVID because it modulates mitochondrial respiration, normalises dysregulated mTOR activity, reduces chronic inflammatory responses, supports vascular function, improves gut microbiome health and provides neuroprotective and epigenetic benefits (23–25). Although Metformin has been found to lower the risk of long COVID with early treatment of the acute infection (within 7 days of a positive test result), there are no published trials about the effectiveness of Metformin in treating long COVID (26). Metformin is considered safe to use and most people can tolerate it well, although some can suffer side effects such as diarrhea, nausea, stomach pain and a higher risk of hypoglycaemia among those who do not have diabetes (27). Metformin is easily available and relatively affordable when purchased with a prescription under the PBS scheme in Australia. There are four clinical trials that are currently in progress looking specifically as treatment in long COVID patients (See Table 2).

**Table 2:** Summary of registered clinical trials in progress.

Study ID	Completion (Date)	Protocol reference/Trial title	Target sample size (N)	Intervention and dose	Treatment duration	Follow-up	Comparator	Primary outcome
Low-done Naltrexone (*n* = 3 trials)								
NCT05430152	2025-08-16	Low-dose naltrexone for post- COVID fatigue syndrome: a study protocol for a double- blind, randomised trial in British Columbia. *BMJ Open*. 2024;14 (5):e085272.	160	Low-Dose Naltrexone as a compounded capsule starting at 1 mg/day and increasing up to 4.5 mg/day (by wk. 4)	16 wks	16 wks	Placebo (to look exactly like LDN doses)	Change in Fatigue intensity by 4.7 points over using the Fatigue Severity Scale (FSS)
ACTRN1262300 1,042,639	2025-04-01	Efficacy of Low Dose Naltrexone for the treatment of symptoms of Post COVID-19 Condition	56	Naltrexone Hydrochloride at low doses (low dose naltrexone [LDN], 3-6 mg/day)	12 wks	12 wks	Placebo	DSQ Symptom Inventory Questionnaire (Determine detectable change in symptom presentation and severity)
ACTRN1262400 1,162,505	2029-10-14	Low dose naltrexone for the treatment of Myalgic Encephalomyelitis/Chro nic Fatigue Syndrome (ME/CFS) and long COVID Condition	56	Naltrexone-start 1.5 mg/day and will increase their dose by 1.5 mg/day weekly until their maximum dose is reached (target 4-6 mg/day for 12 wks)	12 wks	12 wks	Placebo	Change in the Transient receptor potential cation channel subfamily M member 3 (TRPM3) function
Antivirals (*n* = 8 trials)								
NCT06055244	2025-05-15	Amantadine Therapy for Cognitive Impairment in Long COVID (AmantadineLC)	60	Amantadine-100 mg 2 × per day	Unknown	16 wks	Placebo	Overall cognitive functioning (self-assessed and objective), anxiety, depression, side-effects
NCT06234462	2025-06-01	A Study of Amantadine for Cognitive Dysfunction in Patients With Long-Covid	30	Amantadine + standard care −100 mg 2 × per day	4 wks	6 wks	Standard of care = PT, OT, SLP, provider counselling, and/or pharmacologic interventions	Cognitive functioning (R BANS), FAS, Trails A& B, Digit vigilance test (DVT), cognitive subscale of modified fatigue impact scale (MFIS)
NCT05668091	2024-09-08	A Decentralized, Randomized Phase 2 Efficacy and Safety Study of Nirmatrelvir/Ritonavir in Adults with Long COVID	100	Nirmatrelvir 2 × 150mg tablets 2 × per day + Ritonavir 1 × 100 mg capsule 2 × per day	15 days	28 days for primary	Placebo + Ritonavir (100 mg twice per day)	Primary- Physical Health summary; depression, physical function, pain interference, fatigue, sleep disturbance, and satisfaction with participation in social roles (PROMIS-29)
NCT05823896	2024-11-30	ImPROving Quality of LIFe in the Long COVID Patient (PROLIFIC)	219	Oral nirmatrelvir (300 mg + ritonavir) (100 mg) 2 × per day	15 days	Up to 90 days	Placebo/ritonavir (100 mg tablet of ritonavir twice per day)	Quality of life (EQ-5D-5L VAS scale), other secondary outcomes such as; hemodynamic response over time (active standing test), Composite Autonomic symptom score (compass31)
NCT05595369/NCT05965726	2025-03-13	RECOVER-VITAL: Platform Protocol to Measure the Effects of Antiviral Therapies on Long COVID Symptoms (RECOVER-VITAL)/ RECOVER-VITAL: Platform Protocol, Appendix to Measure the Effects of Paxlovid on Long COVID Symptoms (RECOVER-VITAL)	964	Arm 1; Paxlovid 25 days (nirmatrelvir 300 mg, ritonavir 100 mg) bid × 25 Days	25 or 15 days	Up to 90 days	Placebo control (ritonavir 100 mg + nirmatrelvir matching placebo for 25 days)	Change in Cognitive function (PROMIS cognitive function-8a), change in autonomic dysfunction (OHQ- orthostatic hypotension questionnaire), Change in exercise intolerance symptoms (DSQ-PEM), Other secondary outcomes such as serious adverse events
				Arm 2; Paxlovid 15 days (nirmatrelvir 300 mg and ritonavir 100 mg) bid x 15 days then ritonavir 100 mg bid plus nirmatrelvir matching placebo × 10 days				
NCT06316843	2024–10	Valacyclovir Plus Celecoxib for Post-Acute Sequelae of SARS-CoV-2 (PASC)	59	Arm 1–1,500 mg valacyclovir + 200 mg celecoxib −2 x per day	10 wks	12 wks	Placebo (placebo capsules taken 2 × per day)	Fatigue assessed with PROMIS Fatigue7a instrument
				Arm 2–750 mg valacyclovir + 200 mg celecoxib-2 × per day				
NCT06161688	2025-12-31	Ensitrelvir for Viral Persistence and Inflammation in People Experiencing Long COVID (PREVAIL-LC)	40	Ensitrelvir oral capsule- 375 mg on day 1, followed by 125 mg daily for 4 additional days	5 days	Baseline, 10 days and up to 60 days post study	Placebo	Primary outcome is change in patient reported outcomes such as physical function, anxiety, depression, fatigue, sleep disturbance, social, and pain (PROMIS-29)
NCT06511063	2026–01	Antiviral Clinical Trial for Long Covid-19	90	Arm 1 (300 mg tenofovir) oral capsule 1 × day	90 days	Day 180	Placebo pill (once per day, oral capsule for 90 days)	Health status that contains 5 dimensions of mobility, self-care, usual activities, pain/discomfort, anxiety/depression) (EuroQol 5-Dimension 5-Level (EQ-5D- 5 L) Individuals own rating of overall health 0 -worst to 100- best (Visual Analogue Scale-VAS)
				Arm 2 (300 mg Selzentry) Oral capsule 2 × per day				
Antihistamines (*n* = 1 trial)								
ISRCTN10665760	2024–12	STIMULATE-ICP: A pragmatic, multi- centre, cluster randomised trial of an integrated care pathway with a nested, Phase III, open label, adaptive platform randomised drug trial in individuals with long COVID	1,555	Loratadine 10 mg 1 × day + famotidine-40 mg 1 × day	12 wks (84 days)	12 and 24 wks	No drug (usual care)	Fatigue (FAS-Fatigue assessment scale) at baseline, 12 and 24 wks.
Metformin (*n* = 4 trials)								
NCT06147050	2024–12	Effect of Metformin in reducing fatigue in long COVID in Adolescents (REVIVE)	16	Metformin- 500 mg dose of extended- release formulation twice daily for a period of 30 days	30 days	90 days	Placebo twice daily	Mean pediatric Quality of life Multidimension al Fatigue Scale (PedsQL-MFS)
NCT06128967	2025-05-18	A Multicentre, Adaptive, Randomized, double–blinded, Placebo-controlled study in participants with Long COVID-19: The REVIVE Trial (REVIVE)	1,500	Metformin extended- release oral tablet-750 mg	Unknown	60 days	Placebo/Fluvoxamine Maleate-100 MG	Improvement on Fatigue Severity Score Scale (FSS) 60 days after randomization.
KCT0009342	Unknown	Exploratory double-blind randomized placebo- controlled trial of Metformin and Ursodeoxycholic acid (UDCA) to treat post-acute sequelae of SARS-CoV-2 infection (PASC)	396	Metformin- 500 mg	8 wks	Up to 6 mths	Placebo- 500 mg or Test medication 2- “300 mg Urusa (“urusodeoxy cholic acid”) or Placebo 300 mg	Change in PASC score symptoms
CTIS2024-511580- 28-00	2026-10-01	RECLAIM: an adaptive platform trial for the evaluation of treatments for post-acute sequelae of SARS-CoV-2 infection (PASC)	Unknown	Metformin- oral 1,500 mg (max dose per day) *part of platform trial with Colchicine	12 wks	12 wks	Placebo	Patient- reported physical-health related quality of life (HRQoL)
Vagus Nerve Stimulation (*n* = 1 trial)								
NCT05630040	2024-11-08	Vagus Nerve Simulation for Long-COVID-19	40	Portable VNS device daily	6 wks	Baseline, week 2, week 5, week 8, week 12	Sham VNS device	Composite Dysautonomia Symptom Score (COMPASS 31)
Colchicine (*n* = 4 trials)								
ACTRN126210006378 42	2023-06-02	A_multi-centre trial of colchicine vs. control to improve clinical outcomes in adults with long- SARS-CoV-2(COVID-19)	1,000	Colchicine 0.5 mg tablet twice per day given orally	6 mths	6 mths	Standard care	COVID-19 WHO score, Dyspnoea management questionnaire-30
ISRCTN10665760	2024–12	STIMULATE-ICP: A pragmatic, multi- centre, cluster randomised trial of an integrated care pathway with a nested, Phase III, open label, adaptive platform randomised drug trial in individuals with long COVID	1,555	Colchicine 500 mcg taken twice daily by mouth (part of platform trial)	12wks (84 days)	Up to 24 wks	Control (no drug)	Fatigue (FAS-Fatigue assessment scale) at baseline, 12 wks and 24 wks
CTRI/2021/11/038234	Unknown	Colchicine to reduce coronavirus disease-19- related inflammation and cardiovascular complications in high-risk patients post-acute infection with SARS-COV-2-a study protocol for a randomized controlled trial. *Trials*. 2024;25 (1):378.	350	Colchicine 0.5 mg once daily (< 70 kg) or twice daily (> = 70 kg)	26 wks	52 wks	Matched Placebo for 26 wks	Distance walked in 6 min at 52 wks from baseline
CTIS2024-511580-28-00	2026-10-01	RECLAIM: an adaptive platform trial for the evaluation of treatments for post-acute sequelae of SARS-CoV-2 infection (PASC)	Unknown	Colchicine (Up to 1 mg/day)	12 wks	12 wks	Placebo	Patient- reported physical-health related quality of life (HRQoL)
Biologics and Monoclonal antibodies (*n* = 3 clinical trials)								
NCT05877508	2025-07-31	Anti-SARS-CoV-2 Monoclonal Antibodies for Long COVID (COVID-19) (outSMART-LC)	36	AER002–1200 mg administered once by IV (intravenous infusion)	Once	Up to 1 yr	Placebo infusion by IV	Change in Patient- Reported Outcomes Measurement Information System (PROMIS)-29 Physical Health Summary Score from Baseline and Day 90
ISRCTN46454974	2025–12	A research trial to find out if tocilizumab helps adults with Long Covid feel better	152	Tocilizumab-162 mg subcutaneous injection (body weight <100 kg 162 mg fortnightly/body weight ≥100 kg 162 mg weekly for 12 wks)	12 wks	12 wks	Subcutaneous placebo for 12 wks	Health-related quality of life
NCT05926505	2025–08	Safety and Efficacy of Anakinra Treatment for Patients with Post-Acute Covid Syndrome	182	Anakinra- 149 MG/ML (Prefilled Syringe Kineret) Anakinra is injected subcutaneously as 100 mg once daily for 4 wks.	4 wks	Up to 2 years	Placebo- Placebo is injected subcutaneously once daily for 4 wks.	Score of PACS progression reversal
IVIg (*n* = 2 trials)								
NCT06305780/NCT06305793 (Appendix in Supplementary material)	2026–03	Randomized Trial of the Effect of IVIg Versus Placebo on long COVID Symptoms.	380	IVIg + coordinated care, IVIg + usual care, IVIg (Gamunex)- 2 g/kg monthly for 9 months (36 wks)	9 mths	End of 12 mths	IVIg placebo + coordinated care Ivabradine + coordinated care, IVIg placebo + usual care, Ivabradine + Usual care, Ivabradin placebo + usual care	Change in Orthostatic Hypotension Questionnaire (OHQ)/Orthostatic Intolerance Questionnaire (OIQ) Composite Score
NCT05350774	2025-12-15	Immunotherapy for Neurological Post-Acute Sequelae of SARS- CoV-2. clinicaltrials.gov. 2022	45	IVIg- 0.4 g/kg/day for 5 days	5 days	2 wks	Placebo	Proportion of participants with a clinically meaningful change in Health Utilities Index Mark 3 (HUI3) after receiving either IVIg or placebo at Week 2.
Co-enzyme Q10 (*n* = 1 trial)								
NCT05373043	2028-10-31	Long-term COVID and rehabilitation	300	Exercise + mitoquinone (synthetic form of coenzyme Q10) unknown dose	Unknown	4 yrs	Exercise + Placebo	Change in flow mediated dilation (FMD), change in microvascular function (using passive leg movement-PLM), change in cerebral vascular endothelial function (using breath hold acceleration index-BHAI)

ACTRN, Australian New Zealand Clinical Trials Registry number; COMPASS-31, Composite Autonomic Symptom Score; CTIS, Clinical Trials Information System (EU); CTRI, Clinical Trials Registry of India; DSQ, DePaul Symptom Questionnaire; DSQ-PEM, DePaul Symptom Questionnaire; Post-Exertional Malaise; EQ-5D-5L, EuroQol 5-Dimension 5-Level; FAS, Fatigue Assessment Scale; FSS, Fatigue Severity Scale; HUI3, Health Utilities Index Mark 3; ISRCTN, International Standard Randomised Controlled Trial Number; IVIg, Intravenous immunoglobulin; KCT, Korean Clinical Trial Registry identifier; LDN, Low-dose naltrexone; MFIS, Modified Fatigue Impact Scale; mth/mths, Month/months; NAC, N-acetylcysteine; NCT, ClinicalTrials.gov identifier; OHQ, Orthostatic Hypotension Questionnaire; OIQ, Orthostatic Intolerance Questionnaire; PROMIS, Patient-Reported Outcomes Measurement Information System; RBANS, Repeatable Battery for the Assessment of Neuropsychological Status; RCT, Randomised controlled trial; VAS, Visual Analogue Scale; VNS, Vagus nerve stimulation; wk/wks, Week/weeks.

### Nicotine

Theoretically, nicotine could help treat long COVID by competitively binding to nicotinic acetylcholine (nACh) and angiotensin-converting enzyme 2 (ACE2) receptors, displacing SARS-CoV-2 spike proteins and reducing the inflammatory response to the virus (28). Via this mechanism, it is thought that nicotine may improve cognitive function and energy levels amongst many other symptoms of long COVID (27). However, there are no robust clinical trials that have reported on the efficacy of Nicotine as a treatment for long COVID. The only published very low-quality evidence is a case study that involved 4 participants who reported improvements in their symptoms for up to 6 months after using nicotine patches (7.5 mg per day) for 7 days (29). Nicotine patches are readily available without a prescription and are relatively affordable and easy to use. Although nicotine patches provide a slow-release of nicotine, they still present a risk of dependency and can still cause side effects such as nausea and headaches, although serious side effects are rare (30). There are currently no ongoing clinical trials testing the efficacy of nicotine as a treatment for long COVID.

### Vagus nerve stimulation

Vagus nerve stimulation (VNS) is a neuromodulation technique that involves stimulating the vagus nerve to modulate various physiological processes. In the context of Long COVID, VNS is being investigated due to its potential to regulate inflammation and autonomic function, which may address multiple symptoms associated with the condition (31). A pilot RCT focussed on feasibility and acceptability examined the effects of at-home, self-administered transcutaneous auricular vagus nerve stimulation (taVNS) on long COVID symptoms. The study included 13 participants with long COVID who received either active taVNS (*n* = 8) or sham stimulation (*n* = 5) for 15 min twice daily over 2 weeks. The active taVNS group showed significant improvements in fatigue, mood, and cognitive symptoms compared to the sham group (32). The study utilised a commercially available taVNS device that participants could use at home after minimal training. This suggests that the treatment could be relatively accessible and feasible for self-administration. taVNS was well-tolerated, with no serious adverse events observed (30). An RCT testing the effect of taVNS treatment on long COVID has recently been completed but the results are yet to be publicly reported (33).

### Antihistamines

Antihistamines may improve long COVID symptoms by blocking both histamine H1 and H2 receptors and reducing mast cell activation, which is thought to play a role in the pathophysiology of long COVID, thereby reducing brain fog and fatigue. There were no systematic reviews or RCTs investigating the efficacy of antihistamines as a treatment for long COVID. A single non-randomised controlled trial examined the effects of a combination of fexofenadine (180 mg/day) and famotidine (40 mg/day) on long COVID symptoms (34). The study included 14 patients in the treatment group and 13 in the control group. After 20 days of treatment, 29% of treated patients experienced complete resolution of long COVID symptoms, and all treated patients showed significant improvement in evaluated symptoms including fatigue, brain fog, abdominal disorders, and increased heart rate compared to the control group. Antihistamines generally have a well-established safety profile when used as directed and are readily available as relatively inexpensive, over-the-counter medications in Australia (35). The ongoing STIMULATE-ICP trial, which includes a nested drug trial testing famotidine and loratadine, may provide more robust evidence when completed.

### Guanfacine

Guanfacine is a highly selective α2A-adrenergic receptor agonist that is thought to strengthen pre-frontal cortex connectivity and reduce neuro-inflammation and has been used to treat cognitive disorders such as attention deficit hyperactivity disorder (ADHD) (36). It is also thought that guanfacine would be effective as a treatment for cognitive deficits in long COVID (37). A single case report found that eight out of 12 reported improved cognitive abilities when they were with guanfacine (1 mg for the first month, increased to 2 mg after 1 month, if well-tolerated) and 600 mg N- acetylcysteine (NAC) daily (38). Four patients discontinued therapy, two for unspecified reasons and two due to hypotension and/or dizziness, common side effects of guanfacine. Because the use of Guanfacine remains restricted to specific indications and age groups in Australia (people aged 6–17 years as monotherapy for ADHD), there is limited access as a treatment for long COVID symptoms.

### Colchicine

Colchicine is commonly used as an anti-inflammatory medication in the treatment of gout and may help treat long COVID symptoms, in particular pericarditis (inflammation around the heart) and pleuritis (inflammation in the lungs) through a similar mechanism (39). It has been suggested that colchicine may help alleviate long COVID symptoms by suppressing persistent innate immune activation, reducing inflammasome-driven inflammation, improving endothelial function, and limiting fibrotic processes (40). There are no existing studies investigating colchicine as a treatment for long COVID, however there are several trials underway (Table 2). Colchicine is readily available with a prescription and reasonably affordable. It is also generally well tolerated at recommended doses with the most common side effects being gastrointestinal upset (41).

### Nattokinase

Nattokinase is an enzyme produced during the fermentation of soybeans to create ‘natto’, a traditional Japanese food. It has been speculated that Nattokinase may be useful in managing a variety of long COVID symptoms because in-vitro studies have demonstrated that it degrades fibrinaloid microclots and the SARS-CoV-2 spike protein in cell cultures (42, 43). However, there have been no human studies that suggest that it may be beneficial, nor are there any underway or planned for the near future. Nattokinase supplements are readily available over the counter and are relatively inexpensive. Although it is considered to be relatively safe, there are some concerns about combining them with blood thinner medications (44).

### Intravenous immunoglobulins

Intravenous immunoglobulins (IVIg) are derived from donor human plasma and are used to treat antibody deficiencies and auto-immune conditions. Because IVIgs are thought to modulate immune responses, they may be useful in managing a variety of long COVID symptoms (45). Although there are no RCTs investigating the effect of IVIg on long COVID, there are a few very small observational studies that report a positive effect on symptoms (46–48). IVIg is not currently available for people with long COVID in Australia and is very costly if accessed privately. Headache is a minor and relatively common side effect. Other less common side effects include chills, flushing, tiredness, stomach pain, fast heartbeat, and muscle pain (49, 50). There are currently two RCTs in progress testing the efficacy of IVIg for long COVID (Table 2).

### Biologics and monoclonal antibodies

Monoclonal antibodies (mAbs) were originally created and used as treatments for acute COVID infections. However, they could potentially treat a variety of long COVID symptoms by clearing SARS-CoV-2 virus reservoirs by targeting the spike protein of the virus and reducing inflammation by modulating the immune response to the virus (51). A small RCT did not find any significant differences between participants receiving mAb treatment in the form of weekly subcutaneous injections of 700 mg of leronlimab and a placebo group after 8 weeks of treatment (52). mAbs are not approved for treatment of long COVID in Australia, nor are they readily available and their cost is unknown. mAbs have been used to treat a variety of diseases including cancer, safely at least in the short term, with mild side effects such as headaches and nausea (53). However, mAbs treatment does have the potential to set off an anaphylactic reaction and further research is needed to understand their safety profile (54). There are three RCTs underway testing various mAbs (Table 2).

### Coenzyme Q10

Coenzyme q10 (CoQ10) is a supplement that has been widely used for a range of clinical applications and may be a potential treatment for long COVID fatigue because of its role as an antioxidant and in cellular energy function (55). However, the evidence for its efficacy is weak based on data from two RCTs that did not report any benefit from CoQ10 supplementation (56, 57). CoQ10 is relatively safe, readily accessible, and relatively affordable in Australia (58). An RCT that is due to be completed in 2028 may help add to the existing evidence base for CoQ10.

### Multi-component intervention packages

Multi-component intervention packages combine core treatment elements like symptom management (e.g., pain), sustainable increases in physical activity, sleep regulation and pacing, and optional modules addressing issues such as managing breathlessness, mood or cognition. They are usually tailored to the individual’s symptoms and are delivered by a multidisciplinary team often including physical therapists, psychologists and a general practitioner, each of whom focus on one or more symptoms. Evidence from five RCTs suggests that multicomponent interventions can be safe and effective at improving overall quality of life and dyspnoea in individuals with long COVID. There is no strong evidence to suggest that they improve other physical, mental or cognitive symptoms (59–64). However, access to these packages remains limited in Australia, due to lack of coordinated funding. Partial support via reimbursements is available through Medicare under specific care plans for mental and allied health.

## Exercise training

Most of the exercise training interventions for long COVID consist primarily of aerobic and/or strength training with some also incorporating flexibility and balance. In total, 16 RCTs looked specifically at exercise training in long COVID patients (aerobic training k = 3, strength training k = 3, combined exercise k = 8 and pilates k = 2), demonstrating that exercise interventions can be effective in improving fatigue, quality of life, physical performance and mental health in individuals with long COVID (65–80). While individualised, symptom-titrated and supervised programs, appear to be more effective, some authors highlight the challenges associated with implementing exercise interventions within this population. For example, post-exertional malaise which involves a worsening of symptoms following physical exertion can be a major barrier for people with long COVID (81). Exercise is considered to be generally safe, accessible, and low-cost, with most forms easily performed at home and a low risk of adverse events when properly supervised.

## Discussion

This narrative review provides an overview of the available evidence for several potential long COVID treatments that are being considered for future clinical evaluation. In line with recent reviews, the findings highlight the heterogeneity of interventions, ranging from dietary supplements and rehabilitation to complementary therapies. The findings also reinforce the persistent uncertainty around long COVID treatments, despite a rapidly evolving body of evidence. Only six out of 14 of the prioritised treatments have any long-COVID specific RCT-based evidence. The remainder rely on biological plausibility or low quality evidence (case studies).

The treatments reviewed included a range of pharmacological and non-pharmacological approaches aiming to alleviate either symptoms or targeting the underlying biological mechanisms of long COVID. The most common mechanism of action across treatments for long COVID appears to be the reduction of inflammatory pathways and viral reservoirs which might suggest that other treatments that have similar effects may be useful (4). Furthermore, treatments that reduce the viral load early in the infection may also be beneficial and considered as a preventative measure (82). However, the uncertainty around natural history and prognosis of long COVID is a very pertinent and as yet unanswered question which has implications for treatments and management decisions (83).

For clinicians, this research offers a timely overview of the current status of the available evidence for treatments prioritised by clinicians and people with lived experience. Although some treatments such as exercise training show promise and seem feasible and acceptable, there is insufficient high-quality evidence to make informed recommendations for clinicians and people living with long COVID. For funders, researchers and other stakeholders, this review highlights critical evidence gaps, including the need for randomised clinical trials that investigate treatments that are acceptable to and aligned with lived experience.

## Limitations

Although the systematic reviews and RCTs were identified via a systematic search, other study designs were identified through a more pragmatic search to identify the best available, most recent evidence. Furthermore, the interventions summarised were chosen by clinicians and people with lived experience based on their clinical relevance and acceptability. Consequently, this review does not provide a comprehensive list of all potential long COVID treatments. It must also be noted that findings are not intended to be used as clinical guidance – this would require a more robust review of the evidence, and a multidisciplinary and systematic consideration of the clinical and patient context using well established methods to assess the quality of the evidence.

Finally, the overall quality of evidence is very low, due to the lack of high quality RCTs and systematic reviews, underscoring the need for further rigorously designed, well-powered clinical trials.

## Conclusion

In this review, we summarised the evidence for, acceptability, feasibility and safety for 14 stakeholder-prioritised treatments for long COVID. Only six of these have any RCT evidence and most are supported by limited or indirect data. The next step is well-designed trials that use standardised patient centered outcomes, ensure adequate follow-up and consider implementation. This review supports the development of such trials rather than providing guidance in clinical settings.

## Footnotes

1
https://clinicaltrials.gov/

2
https://trialsearch.who.int/
