# Supplementary material for: Candidate treatments for long COVID: a narrative review of expert and patient-driven priorities
Source: Front Med (Lausanne). 2026 Feb 27;13:1734600. doi: 10.3389/fmed.2026.1734600 (PMC12982061; doi:10.3389/fmed.2026.1734600)
Supplement: Supplementary file 1 [file Supplementary_file_1.docx]

**Appendix A: Search terms for the living database**

**PubMed**

("Post-Acute COVID-19 Syndrome"[Mesh] OR "COVID-19/complications"[Majr] OR "post covid"[tiab] OR "post-covid"[tiab] OR postcovid[tiab] OR "post coronavirus"[tiab] OR postcoronavirus[tiab] OR "long covid"[tiab]  OR "Long-covid"[tiab] OR “COVID-19 survivors”[tiab] OR “COVID-19 related”[tiab] OR “history of COVID-19”[tiab] OR “history of COVID”[tiab] OR “Subacute COVID-19”[tiab] OR “after COVID-19”[tiab] OR “subacute COVID-19”[tiab] OR “after covid”[tiab] OR "After SARS"[tiab] OR "Post-Acute Coronavirus"[tiab] OR PASC[tiab] OR ((Post-discharge[tiab]) AND ("Covid-19"[ti])) OR (History[tiab] AND "COVID-19 infection"[tiab]))

AND

(“Randomized controlled trial”[pt] OR “controlled clinical trial”[pt] OR randomized[tiab] OR randomised[tiab] OR placebo[tiab] OR randomly[tiab] OR trial[tiab] OR trials[tiab])

NOT

(Animals[Mesh] NOT (Animals[Mesh] AND Humans[Mesh]))

NOT

(Editorial[pt] OR Meta-Analysis[pt] OR “Observational Study”[pt] OR “Systematic Review”[pt] OR “Case Report”[ti] OR “Case series”[ti] OR Meta-Analysis[ti] OR “Meta Analysis”[ti] OR “Systematic Review”[ti])

**Cochrane**

([mh "Post Acute COVID 19 Syndrome"] OR [mh "COVID 19"/CO] OR "post covid":ti,ab OR post-covid:ti,ab OR postcovid:ti,ab OR "post coronavirus":ti,ab OR postcoronavirus:ti,ab OR "long covid":ti,ab OR Long-covid:ti,ab OR "COVID-19 survivors":ti,ab OR "COVID-19 related":ti,ab OR "history of COVID-19":ti,ab OR "history of COVID":ti,ab OR "Subacute COVID-19":ti,ab OR "after COVID-19":ti,ab OR "subacute COVID-19":ti,ab OR "after covid":ti,ab OR "After SARS":ti,ab OR "Post-Acute Coronavirus":ti,ab OR PASC:ti,ab OR ((Post-discharge:ti,ab) AND (Covid-19:ti)) OR (History:ti,ab AND "COVID-19 infection":ti,ab))

**Embase (Elsevier)**

('long COVID'/exp/mj OR 'post covid':ti,ab OR post-covid:ti,ab OR postcovid:ti,ab OR 'post coronavirus':ti,ab OR postcoronavirus:ti,ab OR 'long covid':ti,ab OR Long-covid:ti,ab OR 'COVID-19 survivors':ti,ab OR 'COVID-19 related':ti,ab OR 'history of COVID-19':ti,ab OR 'history of COVID':ti,ab OR 'Subacute COVID-19':ti,ab OR 'after COVID-19':ti,ab OR 'subacute COVID-19':ti,ab OR 'after covid':ti,ab OR 'After SARS':ti,ab OR 'Post-Acute Coronavirus':ti,ab OR PASC:ti,ab OR ((Post-discharge:ti,ab) AND (Covid-19:ti)) OR (History:ti,ab AND 'COVID-19 infection':ti,ab))

AND

(random* OR factorial OR crossover OR placebo OR blind OR blinded OR assign OR assigned OR allocate OR allocated OR 'crossover procedure'/exp OR 'double-blind procedure'/exp OR 'randomized controlled trial'/exp OR 'single-blind procedure'/exp) NOT ('animal'/exp NOT ('animal'/exp AND 'human'/exp))
